# Supplementary material for: A Dynamic Network Approach for the Study of Human Phenotypes
Source: PLoS Comput Biol. 2009 Apr 10;5(4):e1000353. doi: 10.1371/journal.pcbi.1000353 (PMC2661364; doi:10.1371/journal.pcbi.1000353)
Supplement: Text S1 — Supplementary Material (2.33 MB DOC) [file pcbi.1000353.s001.doc]

# Text S1

# A dynamic network approach for the study of human phenotypes

César A. Hidalgo^1, †^, Nicholas Blumm^2, 4^, Albert-László Barabási^2, 3^, Nicholas A. Christakis^4^

^1^Center for International Development, Harvard University, Cambridge, MA, 02138 ^2^Center for Network Science, Dept. of Physics, Biology and Computer Science, Northeastern University, Boston, MA, 02115 ^3^Center for Complex Network Research and Dept. of Physics, University of Notre Dame, Notre Dame, IN, 46556
^4^College of Computer and Information Science, Northeastern University, Boston, MA, 02115
^5^Dept of Health Care Policy and Dept. of Medicine, Harvard Medical School, Boston, MA, 02115
^†^ To whom correspondence should be addressed: cesar_hidalgo@ksg.harvard.edu

Table of Contents

[Data Set 2](#__RefHeading___Toc220255521)

[Data set description 2](#__RefHeading___Toc220255522)

[Number of Patients per Demographic Group 2](#__RefHeading___Toc220255523)

[Percentage of Visits per Patient and Primary Code Network 3](#__RefHeading___Toc220255524)

[Quantifying Comorbidity Strength 4](#__RefHeading___Toc220255525)

[φ-Correlation 4](#__RefHeading___Toc220255526)

[Relative Risk 4](#__RefHeading___Toc220255527)

[Box 1: Quantifying Disease Associations 6](#__RefHeading___Toc220255528)

[Resource Files 7](#__RefHeading___Toc220255529)

[File structure 8](#__RefHeading___Toc220255530)

[Visualizing the Disease Network 8](#__RefHeading___Toc220255531)

[PDN constructed using ICD9 codes at the 5 digit level 10](#__RefHeading___Toc220255532)

[Male Female PDN centered in Diabetes 11](#__RefHeading___Toc220255533)

[The Evolution of Patient Illness within the PDN 12](#__RefHeading___Toc220255534)

[Connectivity and Lethality 16](#__RefHeading___Toc220255535)

[Multivariate Analysis 17](#__RefHeading___Toc220255536)

[Directionality Analysis 18](#__RefHeading___Toc220255537)

[References 19](#__RefHeading___Toc220255538)

## Data Set

### Data set description

We used MedPAR records for 1990-1993 where the dates and reasons for all hospitalizations were reported in ICD-9-CM format. Each record consists of the date of visit, a primary diagnosis and up to 9 secondary diagnosis, all specified by ICD9 codes of up to 5 digits. The first three digits specify the main disease category while the last two are used to give additional information about the disease. In total, the ICD-9-CM classification consists of 657 different categories at the 3 digit level and 16,459 categories at 5 digits.

We distinguish four main groups in the data set given by (Males = M, Females=F, White=W, Black = B)

### Number of Patients per Demographic Group

|  | M | F | M+F |
| --- | --- | --- | --- |
| W | 4910362 (37.66%) | 6835054 (52.42%) | 11745416 (90.08%) |
| B | 386663 (2.97%) | 596432 (4.57%) | 983095 (7.54%) |
| B+W | 5297025 (40.62%) | 7431486 (56.99%) | 12728511 (97.62%) |
| B+W+Other | 5440490 (41.72%) | 7598529 (58.28%) |  |
| Other (Hispanic+Asian+Native American+Other) | | | 310507 (2.38%) |
| Total | | | 13039018 (100%) |

### Percentage of Visits per Patient and Primary Code Network

An alternative approach to build the disease network would be to consider only primary diagnoses. We have decided not to take that option because of the fact that most individuals (44.8%) have only one hospital visit and would therefore not contribute to the construction of a disease network in which only primary diagnoses were considered. Furthermore, 23% of the patients in our data have only two hospital visits and would therefore contribute, at most, given that these diagnoses are different, to only one comorbidity observation. Hence concentrating on the primary diagnosis will result not only in the elimination of most of our data but would produce a selection bias towards those conditions affecting people with several hospital visits. We believe however that creating a network of primary diagnoses would be an interesting alternative given a data set with more longitudinal resolution.


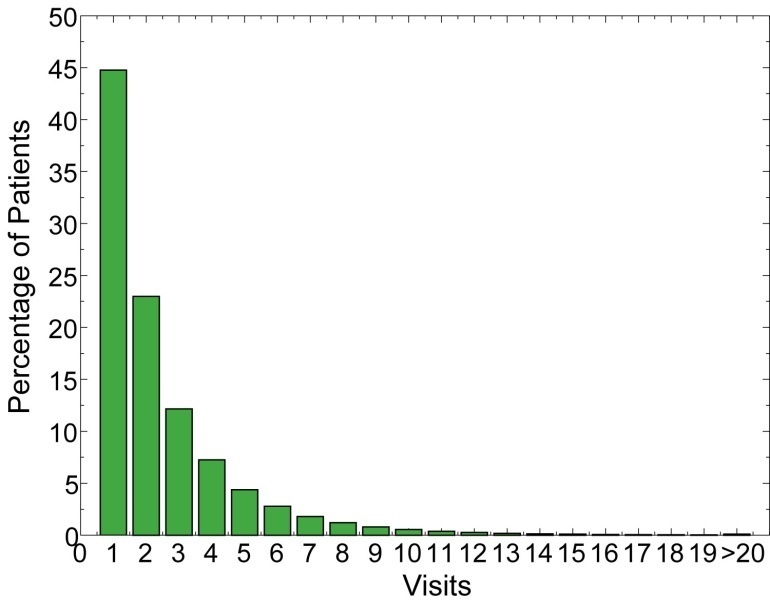


*Figure S 1 Percentage of visits per patient*

## Quantifying Comorbidity Strength

### φ-Correlation

We can quantify the strength of comorbidities by calculating the correlation coefficient associated with a pair of diseases as:


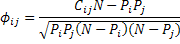


Where *C_ij_* is the number of patients affected by both diseases, *N* is the total number of patients in the studied population and *P_i_* is the prevalence of the *i^th^* disease. The *φ* correlation is the Pearson’s correlation for dichotomous variables, i.e. variables which only take 0 or 1 values. [[[1]](#endnote-2)]

We can determine the significance of *φ* ≠0 by performing a *t*-test. This consists of calculating *t* according to the formula:


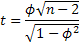


Where *n* is the number of observations used to calculate *φ*. In all of our tables we use *n*=max(*P_i_,P_j_*) << *N*, which represents the most stringent way in which *t* can be calculated given our data, as using *n=N* will produce a larger number of significant links most of which will not necessarily be strong predictors. To determine the level of significance of *t* it is necessary to look for it on a *t*-table which are available online or in most statistics books [Error: Reference source not found]. As a rule of thumb it is important to remember that for *n*>1000 any *t*≥1.96 is significant at the 5% level, whereas for the same *n* any *t*≥2.58 is significant at the 1% level.

### Relative Risk

An alternative way of quantifying the correlation between two variables is to calculate their relative risk. The relative risk is the ratio between the observed co-occurrence and that of a null model. If diseases occurred independently, the number of patients affected by both diseases would be given by:


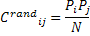


Hence the relative risk of a pair of diseases is given by:


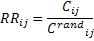


which can also be written explicitly as probabilities as:


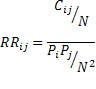


Calculating the significance of the relative risk can be done by using the Katz et al. method to estimate confidence intervals [[[2]](#endnote-3)]. According to their calculations, the 99% confidence interval for the *RR* between diseases *i* and *j* is given by:


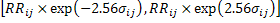


where *σ_ij_* is given by:


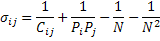


We summarize the pros and cons of each measure in Box 1.

| Box 1: Quantifying Disease Associations We use two different ways to measure the strength of comorbidity associations. We denote *C_ij_* as the number of patients that have been diagnosed with diseases *i* and *j*, *N* as the total number of patients in the population and *P_i_* as the number of patients diagnosed with disease *i*.  φ-correlation Pearson’s correlation for binary variables [[[3]](#endnote-4)] $\varphi_{ij}=\frac{C_{ij}N-P_{i}P_{j}}{\sqrt{P_{i}P_{j}(N-P_{i})(N-P_{j})}}$ (1) **Pros**: Good at discerning associations between diseases with similar prevalence. **Cons:** Values are always low for diseases with extremely different prevalence. **Values**: *φ>0* comorbidity is larger than expected by chance *φ<0* comorbidity is smaller than expected by chance **Range:** *φ ~ *[-1,1] for diseases with similar prevalence. *φ ~ * (P</P>)^1/2^ [-1,1] for diseases with different prevalence where *P< =* min*(P_i_,P_j_)* and *P>=*max*(P_i_,P_j_)* |
| --- |
| Relative Risk Fraction between the number of patients diagnosed with both diseases and random expectation based on disease prevalence. $RR_{ij}=\frac{C_{ij}N}{P_{i}P_{j}}$ (2) **Pros:** Intuitive and easy to calculate. **Cons:** Underestimates associations between highly prevalent diseases and overestimates associations involving rare diseases. **Values:** *RR_ij_>1* comorbidity is larger than expected by chance *RR_ij_<1* comorbidity is smaller than expected by chance **Range:** *RR_ij_~*[N/P_i_P_j_,N/P>] where *P>=*max*(P_i_,P_j_)* |

## Resource **Files**

In this publication we make available a large number of comorbidity data in the form of a PDN. The resource provided consists of 18 different files summarizing a PDN constructed by looking at patients from particular demographics and using ICD9 codes at the 5 and 3 digit levels. Table S 1 shows a brief summary of each of these files.

| **PDN File Description** | | |
| --- | --- | --- |
| **File** | **Patients used** | **Number of Links** |
| AllNet5.net | All patients, ICD9 5 digit level | 6088553 |
| AllNet3.net | All patients, ICD9 3 digit level | 291172 |
| MaleNet5.net | All males, ICD9 5 digit level | 3980759 |
| MaleNet3.net | All males, ICD9 5 digit level | 242056 |
| FemaleNet5.net | All females, ICD9 5 digit level | 4882376 |
| FemaleNet3.net | All females, ICD9 3 digit level | 261804 |
| BlackNet5.net | All black patients, ICD9 5 digit level | 2007916 |
| BlackNet3.net | All black patients, ICD9 3 digit level | 186409 |
| WhiteNet5.net | All white patients, ICD9 5 digit level | 4871245 |
| WhiteNet3.net | All white patients, ICD9 3 digit level | 285795 |
| WhiteMaleNet5.net | All white male patients, ICD9 5 digit level | 3775421 |
| WhiteMaleNet3.net | All white male patients, ICD9 3 digit level | 236513 |
| WhiteFemaleNet5.net | All white female patients, ICD9 5 digit level | 4660064 |
| WhiteFemaleNet3.net | All white female patients, ICD9 3 digit level | 256637 |
| BlackMaleNet5.net | All black male patients, ICD9 5 digit level | 1239552 |
| BlackMaleNet3.net | All black male patients, ICD9 3 digit level | 142226 |
| BlackFemaleNet5.net | All black female patients, ICD9 5 digit level | 1557867 |
| BlackFemaleNet3.net | All black female patients, ICD9 3 digit level | 159215 |

***Table S 1*** *PDN master file description*

### File structure

Each of the 18 files has 10 tab delimited columns. The description of each column is given in Table S 2. Disease pairs are sorted in increasing order with the disease with the lowest ICD9 number always taking the leftmost column.

| **PDN File Structure** | |
| --- | --- |
| **Column** | **Description** |
| 1 | Icd9 code disease 1 |
| 2 | Icd9 code disease 2 |
| 3 | Prevalence disease 1 |
| 4 | Prevalence disease 2 |
| 5 | Co-occurrence between diseases 1 and 2 |
| 6 | Relative risk |
| 7 | Relative risk 99% confidence interval (left bound) |
| 8 | Relative risk 99% confidence interval (right bound) |
| 9 | *φ*-correlation |
| 10 | t-test value |

***Table S 2*** *Column description*

## Visualizing the Disease Network

From a visualization perspective, there is a tradeoff between the number of associations included in a network and the clarity with which these associations can be appreciated. For example, for a network in which comorbidities are quantified using *φ, *we can specify a cutoff value *φ^*^*, such that only links satisfying *φ>φ^*^* are kept. For a large *φ^*^ *cutoff, the resulting PDN is very sparse and uninformative; most diseases will be completely disconnected, whereas for low *φ^*^ *cutoffs the visualization of the PDN becomes exceptionally dense. A good visualization can be obtained by looking for a *φ^*^ *in which there is a component containing a large number of nodes but relatively few links. This can be achieved by examining the number of nodes in the largest connected component of the network for varyous *φ^*^*. This point is known as the percolation transition of the network [[[4]](#endnote-5), [[5]](#endnote-6), [[6]](#endnote-7)].

Here we show examples of this exercise for a PDN constructed using ICD9 codes at the 3 and 5 digit levels. Figure S 2 shows how the giant connected component emerges as the cutoff value *φ^*^* decreases for the network constructed using ICD9 codes at the 3 digit level. For *φ^*^ * = 0.1 the PDN is sparse and incomplete, whereas for *φ^*^ * = 0.05 the network becomes too dense to discern any structure. We find that a good visualization can be obtained by choosing *φ^*^* = 0.06. To finish this visualization we checked the *t*-value for all the associations considered and kept all that were found to be statistically significant at the 1% level.

As another example we show the same exercise for the PDN constructed using our data at the 5 digit level (Figure S 3). Once again, a giant connected component emerges at a similar threshold. A worked version of this network is presented as Figure S 4.


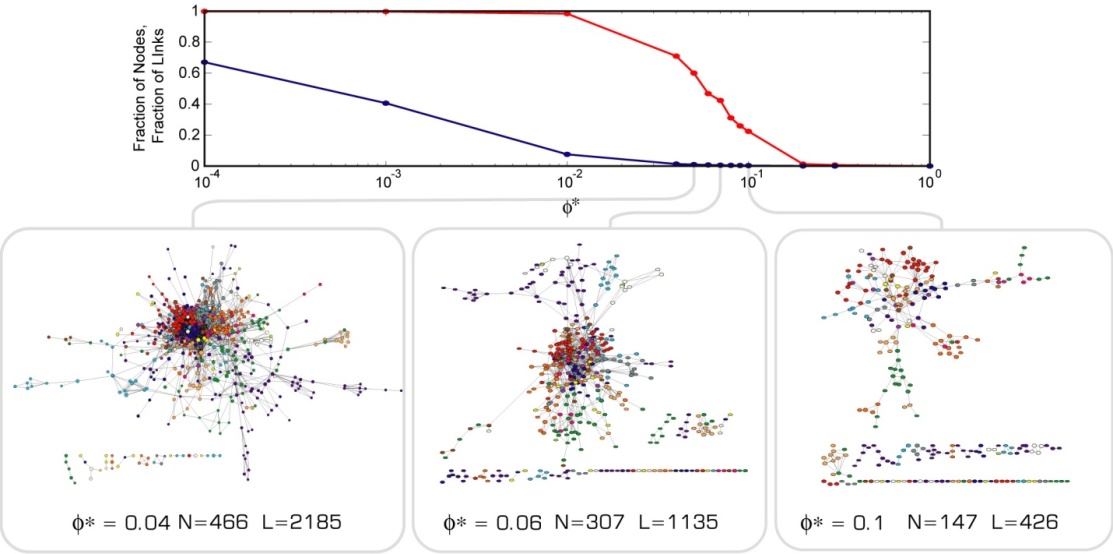


***Figure S 2*** *Number of nodes in the largest connected component of the network as a function the φ threshold for a PDN constructed using ICD9 codes at the 3-digit level. The lower panels show 3 examples of networks at different values of φ^*^. At a given threshold N is the total number of nodes and L is the total number of links in the largest component.*


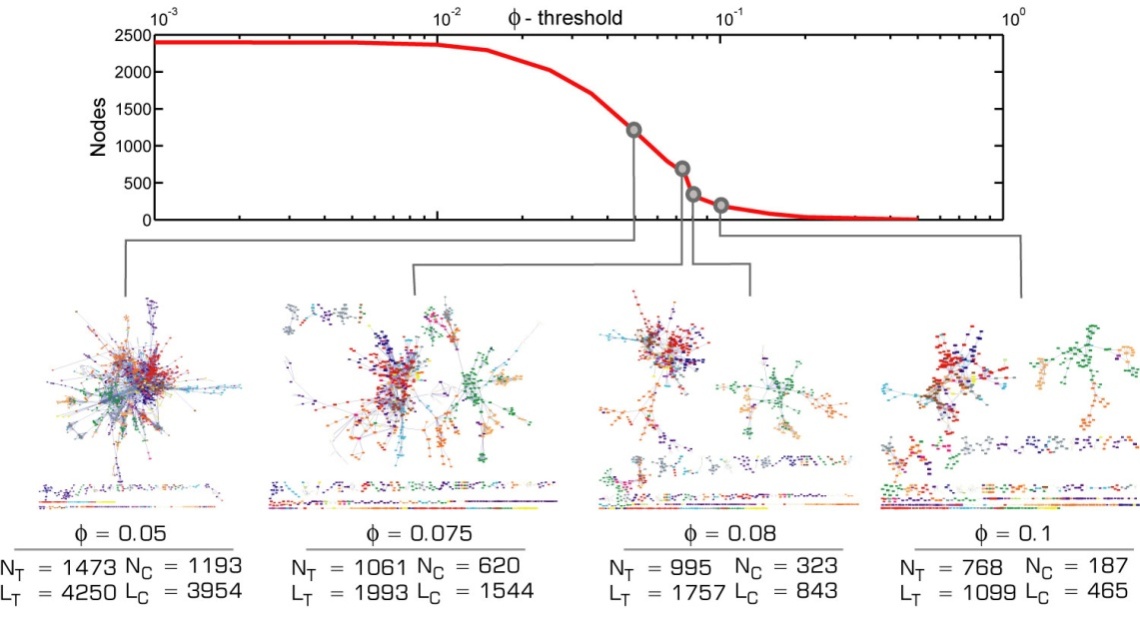


***Figure S 3*** *Number of nodes in the largest connected component of the network as a function the φ threshold. The lower panels show 4 examples of networks at different values of φ. At a given threshold N_T_ is the total number of nodes, L_T_ is the total number of links, N_C_ is the number of nodes in the largest component and L_C_ is the number of links in the largest component.*

### PDN constructed using ICD9 codes at the 5 digit level


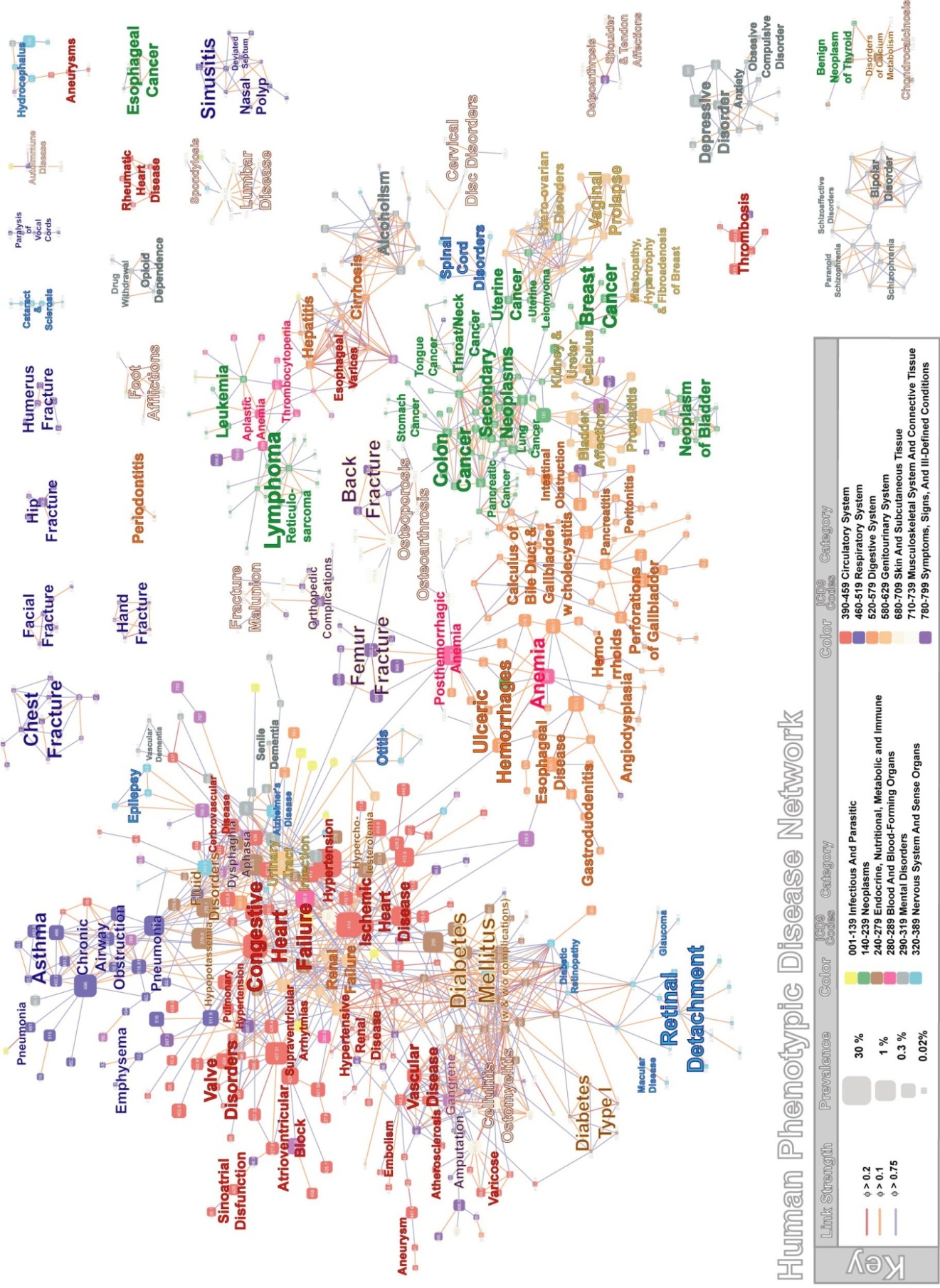


***Figure S 4*** *PDN constructed using ICD9 codes at the 5 digit level and φ-correlation.*

## Male Female PDN centered in Diabetes

Here we present a PDN in which all diseases connected to diabetes with *φ*>0.06. Blue links indicate comorbidities that are strongest among males; whereas red links indicate comorbidities that are strongest among females.


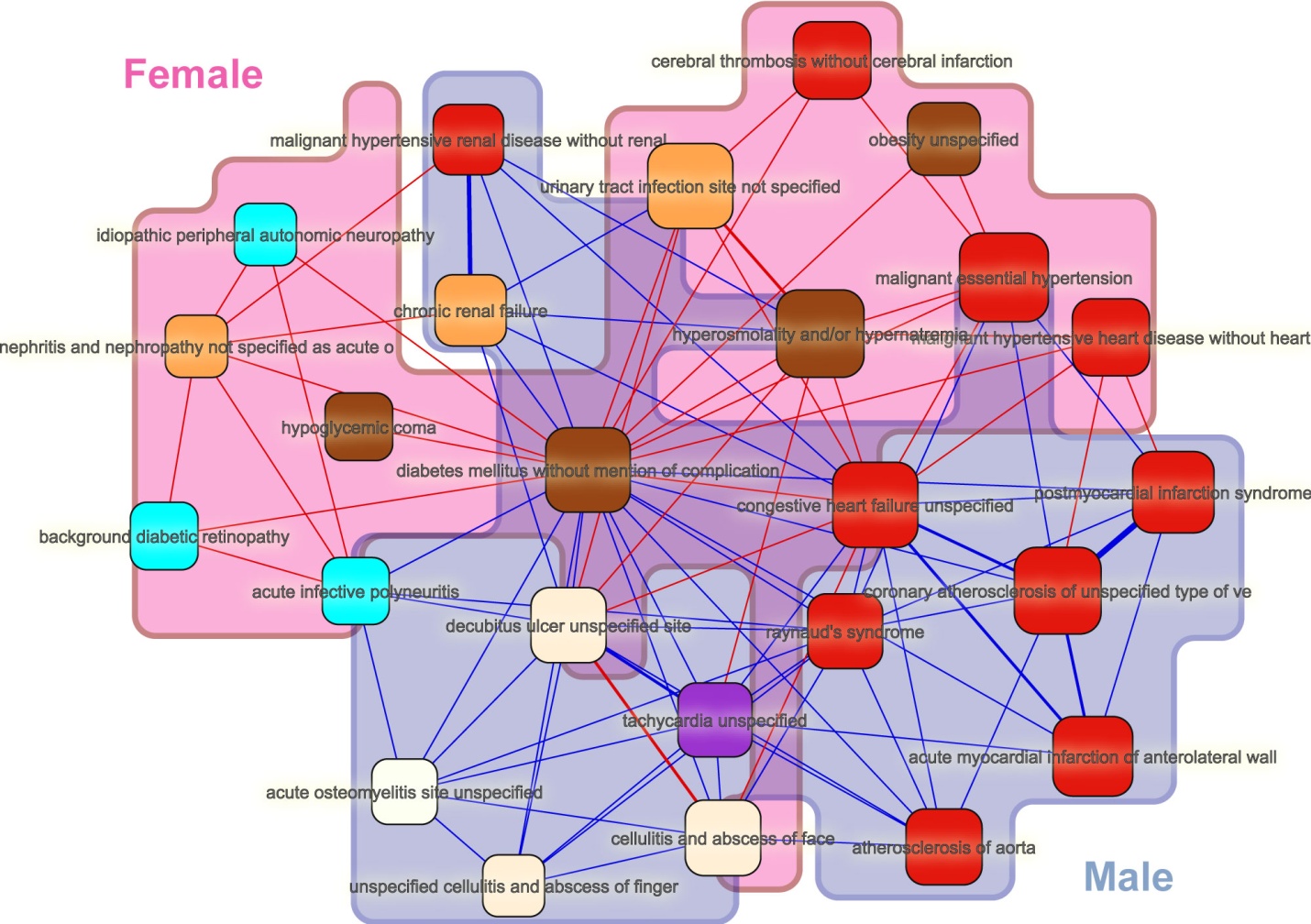


***Figure S 5*** *Comparison between the strength of comorbidities observed in female and male populations.*

## The Evolution of Patient Illness within the PDN

The completeness and detail of the PDN makes it the natural landscape to study the progression of diseases *within* patients across time [[[7]](#endnote-8)]. That is, if the associations presented in Figure S 4 and Figure 2 of the main text are the result of regular disease co-occurrence, we might expect them to represent a predictive substrate in which patient illness evolve. To demonstrate this, we overlay the disease history of three typical patients on the largest cluster of the PDN constructed using *φ* and ICD9 codes at the 5 digit level (Figure S 4), finding that their health evolution is localized in well defined areas of the phenotypic disease network.

In principle, our data set contains more than 2.4 million pairs of disease co-occurring with each other if we consider ICD9 codes at the 5 digit level. The regular way in which illness co-occurs, however, results in a more restricted evolution of patients’ health as structured by the PDN. Patients generally do not develop diseases far removed, in network terms, from the ones they already have. In short, there are niches within the network in which patients experience their illness course, niches which no doubt have complex biological and social origins.

Figure S 6 shows the connections between the diseases diagnosed in the three patients, where direct connections between the diagnoses and nodes lying between any pair of them are shown. The evolution of each patient tells a different story. Patient 1, a 71 year old male, was first diagnosed with Retention of urine (788.2) whereas on his second visit, 160 days later, Prostate Cancer (185) and Urethral stricture (589.9) were reported. Three hundred and forty two days later, in his third visit, bladder cancer (188.8) was diagnosed. The medical history of Patient 2, an 86 year old male, evolved on the diabetes, vascular and osteomyelitic part of the network. He was first diagnosed with Anemia (285.9), Arterial Embolisms of Upper Extremities (444.22), and Diabetic Polyneuropathy (357.2). His second visit revealed Hypertension (401.9) and an ulcer of the lower limb (707.1). These diseases are significantly connected to the previously diagnosed arterial embolism. Later, he developed Gangrene (785.4) and Peripheral Vascular Disease (443.9), which are strongly correlated with the previously diagnosed Ulcer and Embolism. On his fourth visit, an acquired deformity (736.89) and Osteoarthrosis (715.96) were reported. The last visit records peripheral angiopathy (443.81). The evolution of this patient was recorded using 9 different ICD9 codes. Surprisingly, seven of them are connected through 17 of the 1993 possible links with *φ_ij_*>0.075 (χ^2^=175, p=0). Patient 3, a 78 year old female, exemplifies the disease history of an individual whose health evolves in more than one niche. One of these niches shows how she evolved from Pneumonia (486) to Colitis (558.9). Another one shows how cancer spread. On her second visit she was diagnosed with Lung cancer (162.4) and a Secondary neoplasm of intrathoracic Lymph nodes (196.1). Cancer later spread to the lower lobe of bronchus (162.5), bone marrow (198.5) and liver (197.7). Her fourth visit specified that her lung cancer was on the main bronchus (162.2) and her last visit was for Lung cancer (162.9) as well.

These three examples show that the disease progression of patients’ tends to be limited to particular regions of the phenotypic disease network. A statistical generalization of this is presented on the main text.


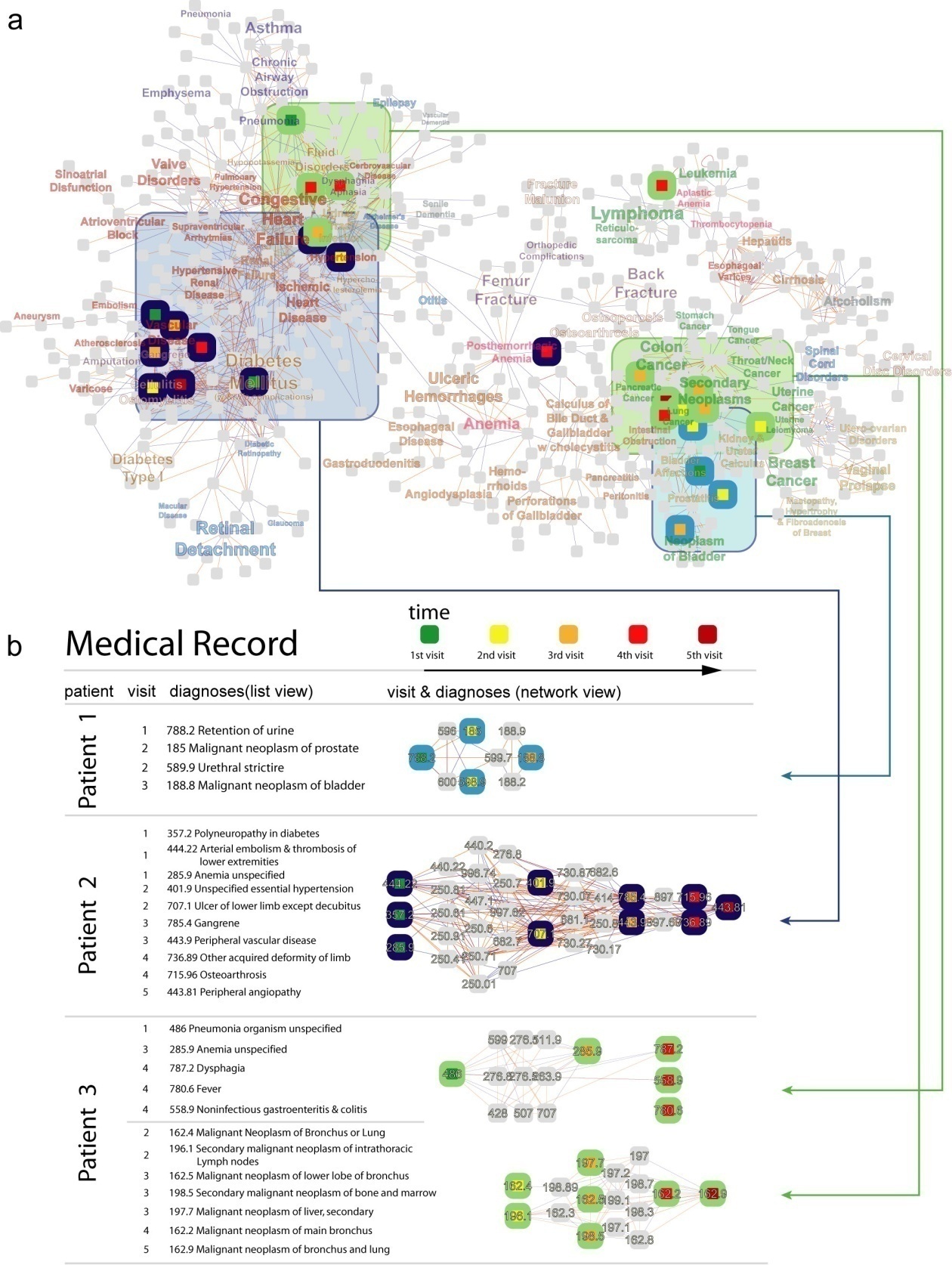
 ***Figure S 6*** *Disease evolution of three patients on the HPDN* ***A.*** *Health evolution of 3 patients on the HPDN. Different patients are indicated by the stroke color of the node: Patient 1, light blue, patient 2, dark blue and patient 3 light green. The visit number is indicated by the fill color of the nodes.* ***B*** *Network medical records of the 3 patients shown in A (same color scheme). The links shown are all those links in Figure S 4 that either connect diseases diagnosed to the patients directly or through another disease in the HPDN.*

## *
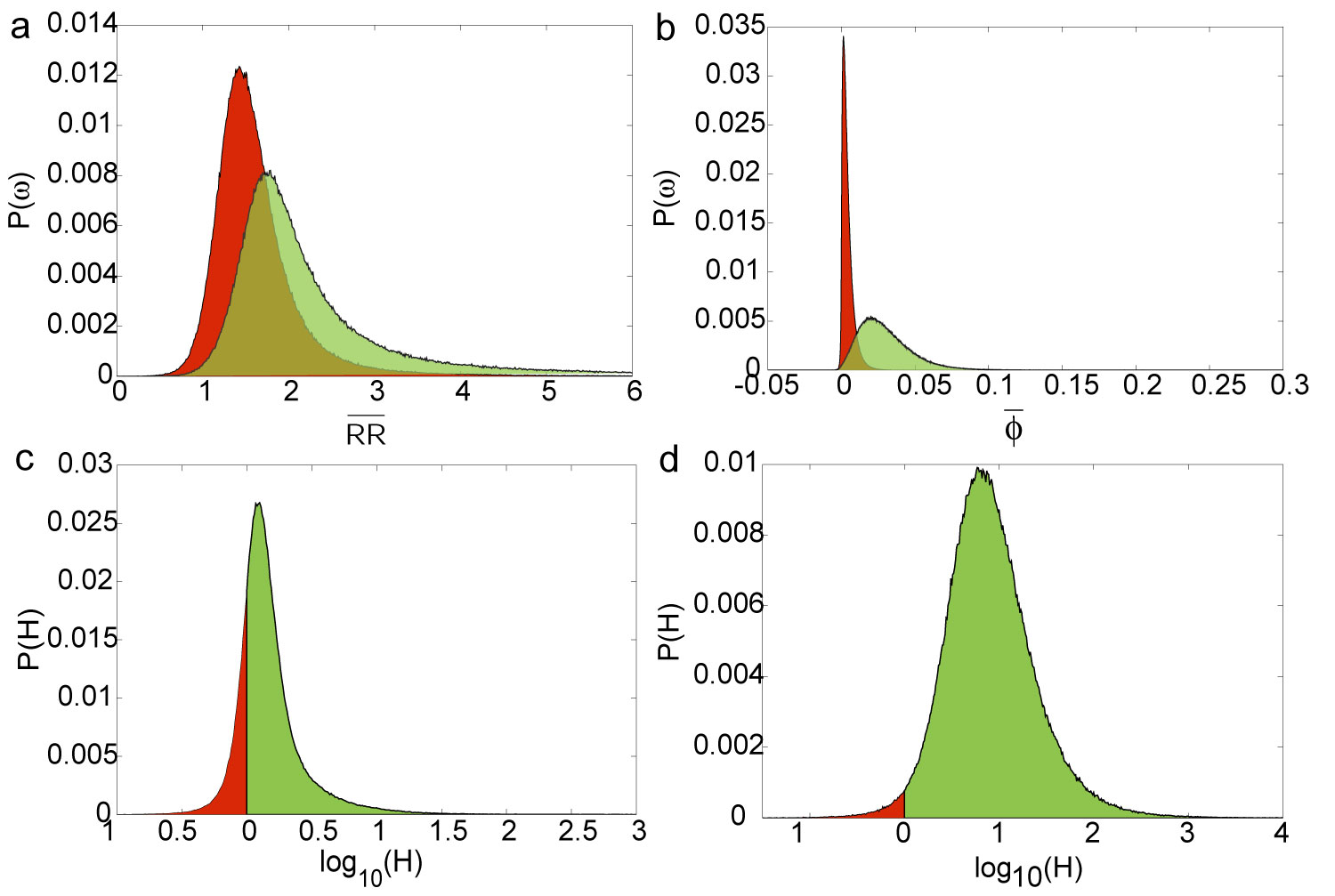
*

*Figure S 7 The same analysis presented in Fig 3 of the paper is shown here starting from a network in which all patients with four diagnosis have been removed. a Average -correlation between diseases diagnosed in the first two and last two visits for the 946,580 patients with 4 visits (green) and when we consider a randomized set of diseases for the first two visits (red). b same as a but for the RR-PDN. c Ratio between the average φ-correlation among diagnoses received by a patient in its first two and last two visits relative to the control case. d. same as c but for the RR-PDN.*

## Connectivity and Lethality

We measure the connectivity of a disease by interpreting the PDN as a weighted network. In a weighted network, the connectivity of a node is the sum of all of its weights. We measure the lethality of a disease by looking at the percent of patients that died 8 years after the disease was first observed in our data set. Table S 3 shows the correlation between connectivity and lethality calculated for the *φ*-PDN and the *RR*-PDN at the 5 digit level split into the 17 disease categories in the ICD9 classification.

| **Icd 9 Category Name** | **Correlation Between log(Lethality) and log(Connectivity)** | | | |
| --- | --- | --- | --- | --- |
|  | *ΣRR* | *p-value* | *Σφ* | *p-value* |
| **001-999 All Diseases and Conditions** | 0.408 | 2.39E-96 | 0.5111 | 2.41E-56 |
|  |  |  |  |  |
| [001-139 Infectious And Parasitic Diseases](http://www.icd9data.com/2007/Volume1/001-139/default.htm) | 0.8908 | 1.69E-12 | 0.7449 | 4.35E-07 |
| [140-239 Neoplasms](http://www.icd9data.com/2007/Volume1/140-239/default.htm) | 0.6378 | 2.51E-27 | 0.5862 | 3.83E-21 |
| [240-279 Endocrine, Nutritional And Metabolic Diseases, And Immunity Disorders](http://www.icd9data.com/2007/Volume1/240-279/default.htm) | 0.71 | 2.65E-18 | 0.6374 | 5.43E-14 |
| [280-289 Diseases Of Blood And Blood-Forming Organs](http://www.icd9data.com/2007/Volume1/280-289/default.htm) | 0.5862 | 2.91E-05 | 0.443 | 2.60E-03 |
| [290-319 Mental Disorders](http://www.icd9data.com/2007/Volume1/290-319/default.htm) | -0.4018 | 2.89E-07 | 0.1464 | 7.19E-02 |
| [320-389 Diseases Of The Nervous System And Sense Organs](http://www.icd9data.com/2007/Volume1/320-389/default.htm) | 0.3197 | 1.52E-06 | 0.6679 | 2.11E-29 |
| [390-459 Diseases Of The Circulatory System](http://www.icd9data.com/2007/Volume1/390-459/default.htm) | 0.5161 | 1.75E-21 | 0.3866 | 6.41E-12 |
| [460-519 Diseases Of The Respiratory System](http://www.icd9data.com/2007/Volume1/460-519/default.htm) | -0.1141 | 0.2146 | 0.4799 | 2.91E-08 |
| [520-579 Diseases Of The Digestive System](http://www.icd9data.com/2007/Volume1/520-579/default.htm) | 0.6292 | 3.53E-30 | 0.4316 | 4.37E-13 |
| [580-629 Diseases Of The Genitourinary System](http://www.icd9data.com/2007/Volume1/580-629/default.htm) | 0.7077 | 3.54E-23 | 0.599 | 2.62E-14 |
| [630-677 Complications Of Pregnancy, Childbirth, And The Puerperium](http://www.icd9data.com/2007/Volume1/630-677/default.htm) | N/A | N/A | N/A | N/A |
| [680-709 Diseases Of The Skin And Subcutaneous Tissue](http://www.icd9data.com/2007/Volume1/680-709/default.htm) | 0.7086 | 8.16E-04 | 0.6725 | 1.58E-09 |
| [710-739 Diseases Of The Musculoskeletal System And Connective Tissue](http://www.icd9data.com/2007/Volume1/710-739/default.htm) | 0.6316 | 6.78E-27 | 0.6271 | 1.15E-25 |
| [740-759 Congenital Anomalies](http://www.icd9data.com/2007/Volume1/740-759/default.htm) | N/A | N/A | N/A | N/A |
| [760-779 Certain Conditions Originating In The Perinatal Period](http://www.icd9data.com/2007/Volume1/760-779/default.htm) | N/A | N/A | N/A | N/A |
| [780-799 Symptoms, Signs, And Ill-Defined Conditions](http://www.icd9data.com/2007/Volume1/780-799/default.htm) | 0.7111 | 1.07E-20 | 0.5937 | 2.96E-13 |
| [800-999 Injury And Poisoning](http://www.icd9data.com/2007/Volume1/800-999/default.htm) | 0.3102 | 5.39E-09 | 0.513 | 6.95E-24 |

***Table S 3*** *Pearson correlation between connectivity and lethality observed in the PDN constructed from all patients using ICD9 codes at the 5 digit level*

## Multivariate Analysis

We study the robustness of our connectivity lethality results by using the linear regression model:


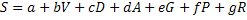


Where the dependent variable *S* is the number of years survived by an individual after the last diagnosis was observed, *a* is a constant term, *V* is the number of hospital visits recorded for that patient, *D* is the total number of diagnosis assigned in all recorded visits, *A* is the age of the patient at the time of the last recorded visit, *G* is the gender (1 male, 2 female), *P* is the logarithm of the connectivity measured using the *φ* PDN and *R* is the logarithm of the connectivity measured using the *RR* PDN.

The coefficients of the regression are:

| **Variable** | **Coefficient** | **Value** | **99% Conf Interval** |
| --- | --- | --- | --- |
| **Constant Term** | *a* | 18.872 | 18.800 18.944 |
| **Number of Visits (V)** | *b* | 0.132 | 0.129 0.135 |
| **Number of diagnosis (D)** | *c* | -0.033 | -0.034 -0.032 |
| **Age (A)** | *d* | -0.0529 | -0.0532 -0.0525 |
| **Gender (G)** | *e* | 0.4822 | 0.4771 0.4872 |
| **Log Σ*φ *(P)** | *f* | -0.613 | -0.625 -0.601 |
| **Log ΣRR (R)** | *g* | -1.875 | -1.892 -1.859 |

***Table S 4*** *Regression coefficients for multivariate analysis between connectivity and lethality*

Table S 4 shows that the correlation between connectivity and the number of years survived by a patient after the last diagnosis is robust to controls for the number of visits, number of diagnosis, age and gender. We notice that more visits are associated with an increase in the number years survived; we also notice that female patients tend to survive longer than males after controlling for all other factors. On the other side, advanced age, more diagnosis and larger connectivities are associated with a decrease in the number of years survived.

## Directionality Analysis

We studied the redundancy between disease connectivity and precedence using the linear model:

*L=a+bK+cΛ**

Where *L* is the fraction of patients that survived, either after 2 or 8 years, *K* is the connectivity of a disease as measured in the *φ*-PDN and *Λ** is the detrended precedence of a disease, as defined in the main text.

For the case in which we study *K* and *Λ** as predictors of the fraction of patients that are deceased 2 years after being diagnosed with a given disease we find:

a=28.9780 with a 99% conf. interval [25.9358 32.0202]
b=0.9402 with a 99% conf. interval [0.5474 1.3330]
c=-0.0831 with a 99% conf. interval [-0.1016 -0.0646]

R^2^ =0.3223 with semipartial correlations (sR):
sR(*K*)=0.0504
sR(*Λ**)=0.1768

For the case in which we study *K* and *Λ** as predictors of the fraction of patients that are deceased 8 years after being diagnosed with a given disease we find:

a=66.3969 with a 99% conf. interval [63.8307 68.9632]
b=1.3496 with a 99% conf. interval [1.0183 1.6809]
c=-0.0530 with a 99% conf. interval [-0.0687 -0.0374]

R^2^ =0.3496 with semipartial correlations (sR):
sR(*K*)=0.14
sR(*Λ**)=0.0971

## References

1. Cohen J, Cohen P, West SG & Aiken LS. Applied Multiple Regression/Correlation Analysis for the Behavioral Sciences. LEA, Mahwah, NJ (2003) [↑](#endnote-ref-2)
2. ? Katz, D., Baptista J., Azen, S.P. and Pike M.C. (1978) Obtaining confidence interval for the risk ratio in cohort studies. Biometrics **34**, 469-474 [↑](#endnote-ref-3)
3. [↑](#endnote-ref-4)
4. Caldarelli G. (2007) Scale-Free Networks: Complex Webs in Nature and Technology,  Oxford University Press, Oxford. [↑](#endnote-ref-5)
5. ? Dorogovtsev SN & Mendes JFF. (2003) Evolution of networks from biological nets to the internet and www, Oxford University Press, Oxford. [↑](#endnote-ref-6)
6. ? Albert R & Barabási A-L. (2002) [Statistical mechanics of complex networks.](http://www.nd.edu/~networks/Publication Categories/03 Journal Articles/Physics/StatisticalMechanics_Rev of Modern Physics 74, 47 (2002).pdf) Review of Modern Physics **74**: 47-97. [↑](#endnote-ref-7)
7. ? Hidalgo CA, Klinger B, Barabasi A-L & Hausmann R. (2007) The product space conditions the development of nations. Science **317**: 482-487. [↑](#endnote-ref-8)
